# The Integration of Biopolymer-Based Materials for Energy Storage Applications: A Review

**DOI:** 10.3390/ijms24043975

**Published:** 2023-02-16

**Authors:** Shrey Dalwadi, Arnav Goel, Constantine Kapetanakis, David Salas-de la Cruz, Xiao Hu

**Affiliations:** 1 Department of Physics and Astronomy, Rowan University, Glassboro, NJ 08028, USA; 2 Department of Biomedical Engineering, Rowan University, Glassboro, NJ 08028, USA; 3 Department of Chemistry, Center for Computational and Integrative Biology, Rutgers University, Camden, NJ 08102, USA; 4 Department of Biological and Biomedical Sciences, Rowan University, Glassboro, NJ 08028, USA

**Keywords:** protein, polysaccharide, biopolymer, battery, lithium ion, zinc ion, capacitor

## Abstract

Biopolymers are an emerging class of novel materials with diverse applications and properties such as superior sustainability and tunability. Here, applications of biopolymers are described in the context of energy storage devices, namely lithium-based batteries, zinc-based batteries, and capacitors. Current demand for energy storage technologies calls for improved energy density, preserved performance overtime, and more sustainable end-of-life behavior. Lithium-based and zinc-based batteries often face anode corrosion from processes such as dendrite formation. Capacitors typically struggle with achieving functional energy density caused by an inability to efficiently charge and discharge. Both classes of energy storage need to be packaged with sustainable materials due to their potential leakages of toxic metals. In this review paper, recent progress in energy applications is described for biocompatible polymers such as silk, keratin, collagen, chitosan, cellulose, and agarose. Fabrication techniques are described for various components of the battery/capacitors including the electrode, electrolyte, and separators with biopolymers. Of these methods, incorporating the porosity found within various biopolymers is commonly used to maximize ion transport in the electrolyte and prevent dendrite formations in lithium-based, zinc-based batteries, and capacitors. Overall, integrating biopolymers in energy storage solutions poses a promising alternative that can theoretically match traditional energy sources while eliminating harmful consequences to the environment.

## 1. Introduction

Batteries and electric storage devices have become ubiquitous in human lives and can be found in cars, phones, and most portable devices. Population growth and increasing demand have propelled advancements to produce cleaner, smaller, and cheaper renewable energy sources [1]. The growing prevalence of implantable energy storage devices in biotechnology calls for increased stability and energy density to maximize the lifespan and minimize the size of the device [2]. Considering the array of uses in the human body (e.g., cochlear implants, implantable defibrillators, and drug delivery systems), an equally diverse array of batteries have been engineered to power these devices [2,3].

Lithium-ion batteries have been utilized extensively due to their high charge density, high coulombic efficiencies, and low self-discharge properties [4]. They are widespread amongst rechargeable portable devices, such as cellphones and computers, as well as electric vehicles [5]. Lithium-ion batteries are composed of a cathode, an anode, a separator, and an electrolyte [6]. The cathodes and anodes carry charge, the separator insulates both the electrodes from each other allowing Li+ ions to flow to each electrode, and the electrolyte carries ions [6,7]. Electrode compositions have been optimized to maximize energy density, as many electrodes contain lithium metal oxides with amounts of nickel, cobalt, and manganese, iron, or titanium [5,7]. Unfortunately, these electrodes suffer from poor electrical conductivity despite high energy density [7]. Furthermore, lithium-ion batteries are susceptible to hazardous failure due to electrochemical instability.

A new frontier in 2-dimensional allotropic materials allows for the development of novel electrode solutions. Two-dimensional materials such as graphene, transition metal dichalcogenides such as molybdenum disulfide (MoS_2_), and transition metal carbides such as titanium carbide (Ti_3_C_2_), enable rapid diffusion of ions, enhancing their effectiveness as electrodes for metal ion batteries [8]. For example, borophene is similar to graphene, but is created using the physical vapor deposition (PVD) technique, utilized by vaporizing boron so that it deposits onto a thin sheet of silver [9,10]. Despite naturally occurring as a metalloid, when boron is in a two-dimensional form as in borophene, it behaves as a highly conductive metal [10]. Borophene has been demonstrated to be an effective anode for lithium batteries without energy dispersion during the charge/discharge process, ensuring a quick charging time and long lifespan [10,11].

Biopolymers pose another option for novel energy material applications. Biopolymers are biodegradable macromolecules composed of repeating units. Natural biopolymers are derived from living matter, such as proteins, polysaccharides, and nucleic acids. Frequently utilized natural biopolymers include silk, hyaluronic acid, collagen, pectin, and gelatin [12,13,14]. Interestingly, their increase in demand stems from both a cost and toxicity standpoint as an alternative to petroleum-based plastics [15,16,17]. Specifically, the manufacturing, usage, and disposal of common plastics such as polystyrene (PS), polypropylene (PP), and polyethylene terephthalate (PETE) contribute to landfill consumptions, higher greenhouse gas emissions, and pollution among other negative effects [18,19]. Despite the significant established infrastructure and thus convenience of fossil-based plastics, biopolymers present a unique solution to enabling sustainability and biodegradability in materials [20,21]. They are further highlighted by their biocompatibility, low production cost, availability, and high control in fabrication [22]. Electronics typically struggle at their end-of-life due to improper disposal resulting in a health and environmental hazard from toxic leakages [23]. This lack of safe degradation originates from relatively stable bonds present in the material. For batteries, this includes potentially toxic elements such as lead, lithium, cadmium, among other metals [24]. Meanwhile, biopolymer-based electrical devices, previously synthesized from materials such as cellulose acetate, silk, chitosan, and pectin all exhibit the unique advantage of biodegradability from both mechanical breakdown and enzymatic cleavage of susceptible bonds via microorganisms [25]. These same functional groups can also promote the customization of biopolymers. For example, Lim et al. utilizes porous carbons with chitosan for lithium-ion batteries to increase efficiency of battery cycles [26]. In addition, biopolymers can demonstrate variable mechanical properties via control of elastic modulus, allowing higher densities of energy storage in previously incapable chemistries [27]. Many biopolymers further exhibit valuable electrical properties such as conductivity and ion conductance [28,29,30].

Based on the aforementioned advantages of biopolymers, many prior studies have focused on energy applications for supercapacitors, rechargeable batteries, solar cells, and fuel cells [31]. Supercapacitors are a promising alternative to traditional batteries; compared with normal capacitors, their higher capacitance allows them to be a potential large storage form for energy. Biopolymer research in this regard is focused on electrode materials with good conductivity, large surface area, and many active sites [32]. Several studies have focused on composite materials integrating graphene with biopolymers [32]. Combinations of this type include binary composites (e.g., graphene with polyindole [33]), tertiary composites (e.g., graphene, silicon dioxide, and polyaniline [34]), and quaternary composites (e.g., graphene, platinum, carbon nanotubes, and polyaniline [35]). Additionally, biopolymers are a promising possibility for innovation in rechargeable batteries. Polyethylene oxide-based lithium metal polymer batteries have improved flexibility and processability but currently, their lower ionic conductivity limits them in comparison with state-of-the-art lithium-ion batteries [36]. Other potential avenues for biopolymer-based rechargeable batteries include composite polymer electrolytes and hybrid inorganic-organic electrolytes [36]. Furthermore, biopolymers have also been found to be an exciting source for improvement in solar cells, specifically in the application of dye-sensitized solar cells. Dye-sensitized solar cells (DSSCs), relying on organic dyes for the conversion of light energy to electrical energy, are a cheaper, lighter alternative to conventional solar cells [37]. Polysaccharide biopolymers have been investigated as electrolytes for DSSCs, with examples including cellulose, agarose, chitosan, starch, carrageenan, with gelatin a non-polysaccharide electrolyte option [38]. Biopolymers have been explored in fuel cell applications as proton and ion exchange membranes. Nafion is a prominent proton exchange membrane currently; however, it faces limitations due to high costs and methanol crossover [39]. Cellulose, chitin, and alginate-based membranes offer lower monetary and environmental costs in comparison with nafion in proton exchange membranes with cellulose and chitin-based membranes showing more promise for anion exchange membrane options [40].

This review further investigates and characterizes the current state of biopolymer applications for advancements in energy storage applications. The diverse array of biopolymers with potential in energy storage advancement and their fabrication methods were described. Relevant theoretical principles that govern such progress in electrochemistry and outline novel paths were also discussed for future work.

## 2. Theory

A battery cell can be constructed with five components: an anode, a cathode, an electrolyte, a separator, and current collectors. The anode and cathode reside on opposite ends of the battery with the separator between them. The empty space in between is occupied by the electrolyte, which provides ion transportation between the anode and cathode. When a lithium battery is discharged, the lithium ions travel through the electrolyte from the anode to the cathode. The flow of positive ions generates free electrons at the anode and stimulates a current. The current collector provides an electrical current for the device with which it interfaces.

A battery is composed of several cells, each with a cell potential (*V*), specific capacity (A·h·kg^−1^), and gravimetric energy density (W·h·kg^−1^). The electric potential of a cell is the difference between the electrical potential of the cathode and the anode, as shown in Equation (1). It describes the work (*W*) required to separate opposing charges in the battery per the amount of charge separated (*Q*).

(1) $$V=V_{cathode}-V_{anode}=\frac{W}{Q}$$

A battery’s capacity (*C*) is a measure of total electrical current a battery can provide (Equation (2)). It is described in Amp-hours and informs its user of how long the battery can provide a current of specified amperage. Capacity can also be reported in coulombs (C; and 1 Ah = 3600 C). The specific capacity of a battery (Equation (3)) describes the battery’s capacity per unit mass and is more useful for comparing capacities of different sizes or materials. A battery’s gravimetric energy density (Equation (4)) is the amount of energy stored in a space per unit mass. It is typically expressed in units of W·h·kg^−1^.

(2) $$C=I\times t_{d}$$

where *C* represents the capacity, *I* represents the current, and *t_d_* represents the time to discharge the battery.

(3) $$Q=\frac{C}{m}=\frac{I\times t_{d}}{m}$$

where *Q* represents the specific capacity of an electrode and *m* represents the mass of the electrode in kilograms.

(4) $$e=\frac{W\times t_{d}}{m}$$

where *e* represents the gravimetric energy density of an electrode.

### 2.1. Cathodes

A cathode is the site where reduction occurs. Likewise, lithium ions would be reduced in the cathode of a lithium-ion battery. Thus, cathodes consist of anions or compounds that are easily able to reduce lithium ions. The most common cathode materials are transition metal oxides, such as LiCoO_2_ and LiNiO_2_, capable of supporting higher valences after releasing the lithium ions [41]. These high-performance cathodes are referred to as 4 V cathode materials because they deintercalated lithium at 4 V vs. Li/Li^+^ [42]. Moreover, transition metals can often support multiple oxidation states, remaining stable in its reduced state when the battery is discharged and its oxidized state when the battery is charged. However, cathode materials must easily withstand phase changes associated with large scale oxidation; crystalline structures are preferred for their resilience to compositional changes [43].

A rechargeable cathode can accept and release ions repeatedly. The more rapidly a cathode can accept lithium ions, associated with the cathode’s surface area, the greater the current the battery is capable of supplying. Resultant reduction in lithium ions in a LiCoO_2_ battery can be represented by Equation (5), which can be applied to other cathode materials as well. LiCoO_2_ is used in this example due to its prevalence. However, other more cost-effective transition metals, such as manganese, nickel, and iron have shown promising potential as alternative cathode materials [44,45].

CoO_2 (s)_ + Li^+^_(aq)_ + e^−^ → LiCoO_2 (s)_ (5)

### 2.2. Anodes

While reduction occurs in the cathode of a battery cell during discharge, oxidation occurs in the anode during charge. The anode can store activated ions in a high energy state; therefore, a good anode can hold ions as densely as possible and in the highest state possible [46]. Theoretically, pure metal such as lithium, would be the most efficient anode; however, it is more practical to use non-metal materials such as graphite to avoid dendrite formation. Equation (6) shows the mechanism of lithium ions with a graphene anode.

LiC_6 (s)_ → Li^+^_(aq)_ + C_6 (s)_ + e^−^ (6)

**Figure 1:**
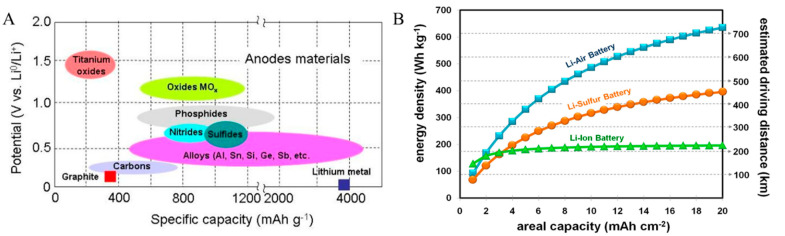
(**A**) The electric potential and specific capacity of various anode materials [49]. (**B**) Plot of energy density (W·h·g^−1^) vs. areal capacity (mA·h·cm^−2^) for three distinct anodes. Energy density is also displayed in terms of a theoretical driving distance if these batteries were used in an electrical vehicle [50]. Reproduced with permissions ((**A**). Copyright AIMS Press 2016 under CC BY 4.0; (**B**). Copyright 2014 Springer Nature).

More recently, researchers have experimented with lithium anodes, planning to utilize new technologies and elevate the efficiency of batteries to their theoretical maximum [47,48]. With a high energy density and low physical density, lithium can store ions without adding exceptional mass to the battery. Other potential anode materials include metal-oxides, phosphides, as well as varying alloys such as aluminum, tin, and silicon [49]. The capacities of some anode materials are displayed in Figure 1A.

As demonstrated in Equation (1), the lower potential (V) for the anode can typically allow for the highest electric potential for the battery cell. In that regard, graphite and lithium metal are both excellent anodes, indicated by reduction potentials against Li/Li^+^ of less than −0.5 V [49,50]. However, lithium boasts a specific capacity of 3860 mA·h·g^−1^, nearly ten times higher than graphite’s 372 mA·h·g^−1^ [50,51].

While lithium metal has a high specific capacity, another important consideration for producing cells with a high energy density is the areal capacity. The areal capacity is described as the capacity per unit electrode area. Typically, a greater areal capacity lends to a greater energy density [50]. For improved energy density, pairing lithium with other mixtures or compounds has shown promising results. Lithium–sulfur and lithium–air anodes both provide significantly increased energy density compared with lithium, as illustrated in Figure 1B.

### 2.3. Electrolytes

Typically, the requirements for an efficient electrolyte are simple: they should remain liquid under operating conditions, should be capable of transporting ions, and should be as cheap and safe as possible [52]. These requirements correspond to physical and chemical properties associated with high chemical stability, high melting points and boiling points over the correct domain, high dielectric constant, low viscosity, high Li-ion conductivity, low electron conductivity, and a limited potential for danger (flammability, explosiveness, and toxicity). Developments in electrolyte materials have been a prominent focus in recent years due to their role in maximizing the current density and the stability of the battery [53].

Briefly, three types of electrolytes used with Li-ion batteries were discussed: non-aqueous electrolytes, aqueous solutions, and polymer electrolytes. Organic solvents are the most common electrolyte found in lithium-ion batteries and are often combined with carbonates. The use of organic compounds manifests in several ways depending on the type of electrolyte. Lithium-ion batteries were primarily discussed in this review because of their nearly unrivaled volumetric energy density; however, solid state batteries, which allow for improved durability and reduced weight, are often used for other batteries [43,53].

Electrolytes behave differently in apolar media than they would in dipolar media, thus highlighting the starkest contrast between aqueous and non-aqueous electrolytes. Dielectric permittivity (*ε*), the measure of a substance’s polarizability, is an important reason for this distinction. The dielectric permittivity of water and the non-aqueous n-hexane are 78 and 1.88, respectively [54]. The high polarity of water can induce the dissociation of organic solutes creating forming ions; strong electrolytes dissociate completely, and weak electrolytes dissociate only partially. However, non-aqueous solutions often have low dielectric permittivity, leading to a hesitance for solutes to dissociate. Values for *ε* can be found using Equation (7), where *D* is the electric displacement field and *E* is the applied electric field.

*D* = *εE* (7)

While aqueous electrolytes can form small ions with ease and abundance, non-aqueous electrolytes are preferred when incorporating large ions [54]. With a lack of polarity in most non-aqueous electrolytes, ion dissociation is governed by the Van der Waals forces (Equation (8)),

*u(r)* = *−β/r^6^* (8)

where *u* is the interaction energy associated with two identical molecules, *β* is correlated with the size of the molecules, and *r* is the radial distance between them. Due to the sixth order of *r*, repulsive and attractive forces between two molecules in a non-aqueous solution greatly weakens with increased distance. Therefore, ions in non-aqueous electrolytes remain close to each other, benefiting from a high energy density [55].

Polymer electrolytes have received recent attention due to increased stability and high ionic conductivity compared with non-aqueous solvents [56]. Research over the past two decades has identified polymer-gel electrolytes capable of reducing the negative effects of crystallinity on ionic conductivity in traditional polymers [57]. However, much research needs to be performed before understanding the most appropriate uses and most efficient compositions of polymer/biopolymer-based electrolytes [58]. As it stands, polymer/biopolymer electrolytes are currently used or researched for solar cells, fuel cells, solid-state batteries, supercapacitors, mobile electronic devices, etc. [56].

## 3. Biopolymer Materials

### 3.1. Silk

**Figure 2:**
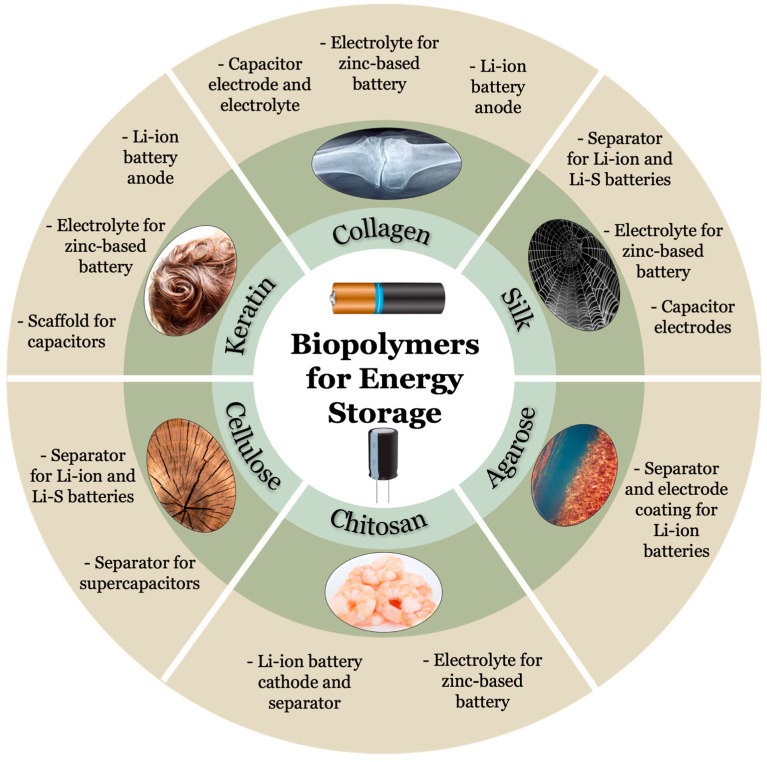
Source and applications for biopolymers commonly utilized for energy storage purposes such as batteries and capacitors. Keratin, collagen, and silk are protein-based biopolymers while cellulose, chitosan, and agarose are polysaccharide-based biopolymers. (Images of biopolymers were obtained with permission from pixabay.com, accessed on 1 December 2022).

Silk is one of the oldest protein-based biopolymers harnessed by mankind, utilized for a variety of functions especially in the textile industry (Table 1) [59]. Silk is produced by silkworm (*Bombyx mori*), orb-weaver spiders, and numerous organisms of the order Lepidoptera as seen in Figure 2 [60]. The structure of silk directly correlates to its unique properties such as biocompatibility, biodegradability, mechanical strength, and precise control in fabrication [61]. Silk is a highly crystalline material, composed of repeating amino acid motifs in its primary structure and compact β sheet regions in its secondary structure [62]. Silk is further broken down into its unique variants, most notably silk I (with helices/coils) and silk II (with β sheets). The distinct crystal formations between silk I (monoclinic unit cell) and silk II (orthorhombic) result in a dimorphic crystalline structure that is a prerequisite for piezoelectric materials [63].

### 3.2. Keratin

Keratin is a protein-based biopolymer known for its robust mechanical properties and self-assembling capability [64]. Keratins are classified as “hard”, stemming from the hair and “soft”, deriving from epithelial tissue. Hard keratins are typically associated with higher contents of the cysteine residue, yielding higher degrees of sulfur-based crosslinking [65]. Thus, as a fibrous protein, keratins are very stable polymers and insoluble. Furthermore, keratins are divided into α-keratins and β-keratins. α-keratins are found to be a tightly coiled right-handed helix with 3.6 amino acids per turn. This tertiary structure is established via hydrogen bonding in the side chains. Generally, α-keratin is the primary material in hair and wool among others. Meanwhile, β-keratins are found as pleated sheets, stabilized via hydrogen bonding between the carboxyl and amino groups in respective residues. β-keratin is usually located in reptiles and birds and is notoriously challenging to extract [66]. For energy applications, keratins are incredibly useful due to the presence of nitrogen atoms that enhance carbon activation for ion transport as well as inherent biopolymer properties such as biodegradability [67].

### 3.3. Collagen

Collagen is a protein-based polymer serving as the primary structural protein component in biological systems [68]. Collagen is known for its fibrous, hierarchical composition that ultimately yields a triple coil helix for the quaternary structure known as tropocollagen. These tropocollagens are then bundled to form collagen fibers. This novel polymer design provides collagen excellent mechanical properties including elasticity and strength [69]. Similar to previous polymers, collagen is rich in various atoms such as nitrogen, oxygen, and especially carbon. This translates into applications for doping in order to further induce electrochemical properties into biopolymers [70]. Interestingly, collagen for electrical applications is often sourced from leather waste, highlighting the importance of reusability in this search for more sustainable energy solutions [71].

### 3.4. Chitosan

Chitosan is a derivative of chitin, sourced from the exoskeleton of many insects, arthropods, shellfish, and even cell walls of fungi as portrayed in Figure 2 [72]. As a polysaccharide-based biopolymer, chitin’s structure is composed of β-1,4-glycosidic linked 2-acetamido-d-glucose and 2-amino-d-glucose units. Chitosan is formed by eliminating acetate through the chemical reaction of the previous polymer [73]. Notably, chitosan is seen as an evolutionary intermediate between cellulose and collagen [74]. However, unlike the latter examples, chitosan has a significantly higher percentage of nitrogen atoms, adding functionality as a chelating agent for electrical applications especially as additives within electrodes [75].

### 3.5. Cellulose

Cellulose, the most abundant biomaterial, is found mainly in plants, algae, and some species of bacteria [76]. Cellulose is a polymer fabricated of 1,4 covalent glycosidic bonded β-D glucose monomers and is insoluble despite its hydrophilic nature [77]. When synthesized in nature, cellulose is typically found as a structure of elementary fibrils which form microfibrils which then form macro cellulose fibers [78]. The cellulose fibers typically arrange as a semicrystalline structure in which regions of ordered chains are interrupted by amorphous regions. Four crystalline allomorphs of cellulose exist, cellulose I, II, III, IV. Cellulose I is fabrictaed of a mix of triclinic (Iα) and monoclinic (Iβ) structures and is obtained naturally [79]. Cellulose II, the most stable form of cellulose, can be obtained from cellulose I by regeneration or mercerization of cellulose I [80]. Cellulose III type I can be obtained from treatment of cellulose I and can be used to form cellulose IV type I; cellulose III type II is produced from cellulose II and can be utilized to form cellulose IV type II [80].

Cellulose can also be modified chemically to form cellulose derivatives with slightly distinct characteristics [81,82,83,84]. Cellulose acetate, produced by replacement of hydroxyl groups in glucose with acetyl groups, can be obtained from agricultural byproducts and does not require further chemical or mechanical treatment [80]. Carboxymethyl cellulose, derived from cellulose by hydroxyl group substitution for carboxymethyl group, is more soluble in water and other organic solvents and is effective for the formation of foams and aerogels [85]. This ease in modification in addition to chemical stability render cellulose a potent option for separators in energy storage options [86].

### 3.6. Agarose

Agarose, a linear biomass-derived polysaccharide, is composed of the repeating monomer units of agarobiose fabrictaed of β-D-galactose and α-3,6-lactose-L-galactose [87]. Agarose is typically obtained by extraction from seaweed as suggested in Figure 2 [88]. Characteristics of note are its hydrophilicity, chemical stability, and electrical neutrality [89]. Agarose may be insoluble in room temperature water, but can dissolve in ninety degree Celsius water, allowing for manipulation without harsh reagents [90]. The ether and hydroxyl functional groups aid in directing ion transfer. Agarose is gelated by self-assembly mainly due to hydrogen bond formation, resulting in a porous structure [91]. Such porosity enables agarose to be an ideal candidate for electrode materials.

**Table 1:** Overview of natural biopolymers as energy storage materials.

Biopolymer	Natural Source	Characteristics	Applications
Silk	Silkworms, spiders [60]	Highly crystalline, biocompatible, biodegradable, high mechanical strength [61].	Separator for lithium ion [92] and lithium sulfur batteries [93].
Keratin	Hair, wool (α), reptiles (β) [66]	Stable, self-assembling, strong mechanical properties [65].	Anode in lithium ion batteries [94], electrolyte in zinc-based batteries [95], scaffold in supercapacitors [96].
Collagen	Leather waste [71]	Fibrous, excellent elasticity and strength [69].	Anode in lithium ion batteries [97], electrolyte in zinc-based batteries [98], separator in supercapacitors [99], electrode in supercapacitors [100].
Chitosan	Exoskeletons, fungi cell walls [72]	Biocompatible, biodegradable, functional as a chelating agent [75].	Additive for cathode and separator in lithium sulfur batteries [101], electrolyte in zinc-based batteries [102].
Cellulose	Plants, tunica, algae, bacteria [80]	High mechanical strength, high degrees of polymerization and crystallinity [82].	Separator in supercapacitors [103] and lithium-ion batteries [104].
Agarose	Seaweed [88]	Chemically stable, electrically neutral [89].	Separator [105] and anode coating material [88] in lithium-ion batteries

## 4. Fabrication and Characterization Methods

Various fabrication methods for energy storage materials (solution casting, electrospinning, chemical vapor deposition, hydrothermal treatment, pyrolysis, and 3D printing) can be used to manufacture membranes, fibers, hydrogels, scaffolds, and more. Discussion of fabrication and characterization methods for integrating biopolymers into energy storage materials is crucial for a well-rounded review of the range of applications biopolymers can reasonably fulfill. For example, the ease of manufacturing, cost effectiveness, and time commitment are each important considerations when evaluating the feasibility of a biopolymer application.

### 4.1. Solution Casting

**Figure 3:**
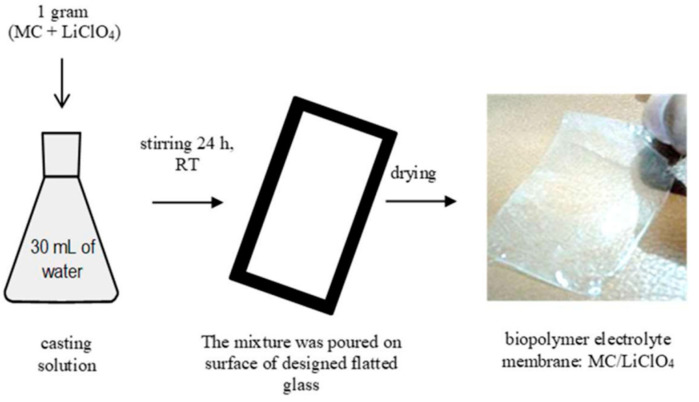
Synthesis of methyl cellulose/lithium perchlorate biopolymer electrolyte membrane [107]. Reproduced with permission (Copyright 2020 ITB Journal Publisher under CC BY 4.0).

Biopolymer electrolyte membranes (BPEs) are synthesized with various biopolymers, including cellulose, chitosan, and starch [106,107]. Chitosan-cellulose-based BPEs were synthesized by mixing the chitosan in hydroxypropyl methylcellulose, and ionic liquids were subsequently added to increase conductivity [106]. Methyl cellulose has been complexed with lithium perchlorate by mixing the solution, casting it on a flat glass surface, and evaporating excess liquid, leaving behind lithium perchlorate membranes. This fabrication process is illustrated in Figure 3.

Similarly, a solution casting technique was utilized to synthesize a starch-resorcinol-formaldehyde (RF) lithium triflate (LiTf) biopolymer electrolyte membrane [108]. Appropriate amounts of RF, ionic salts, and plasticizer’s components were mixed in a solution. The starch solution was prepared separately, gelatinized, cooled, and then the RF solution was mixed in to allow the components to complex. This solution was left to dry to form starch films. Direct incorporation of the biopolymers into the ionic electrolytes is a common and sustainable method for synthesizing BPEs with optimal material properties [106].

### 4.2. Electrospinning

Electrospinning is one of the most versatile and commonly employed techniques for biopolymer manufacturing, especially for electrical applications due to possibility for high porosity and excellent surface to volume ratio. Both traits are imperative to ensure optimal ionic conductivity and transport paths in batteries and capacities [109]. One of the first steps in electrospinning is determining the solvent for the desired biopolymer. Such solvents have been difficult to obtain for certain biopolymers such as cellulose, though alternative forms (such as cellulose acetate) posses more reasonable options [109]. From here, electrospinning follows the same general principle of passing a polymer solution through an electrical field in order to evaporate the solvent and collect the nanofibers on a receiving surface. Often, further treatment is necessary such as annealing and/or reduction [109]. Electrospinning allows for a tunable material blend that can serve various purposes in energy storage devices such as the electrode or separator. Similarly, electrospinning is a highly modifiable process that can effectively control parameters such as fiber diameter, distribution, and formation via altering solution viscosity, operating conditions, and environmental characteristics [110]. All of these enable electrospinning to effectively manufacture biopolymers in the optimal morphology for electrical applications.

### 4.3. Carbonization Methods for Biopolymers

There are many documented approaches for the successful carbonization of biopolymers. The carbonization of biopolymers generally includes the degradation of biopolymer precursors followed by the carbonization of the polymers. Biopolymers are often charring polymers that undergo a series of chemical reaction such as cyclization and crosslinking in order to form a carbon network upon carbonization at high temperatures [111]. Some popular carbonization methods include chemical vapor deposition (CVD) that incorporates nitrogen-rich reactants to form a nitrogen doped graphene coating over a given support with a metal catalyst [112]. CVD is also a tunable method based on the flow rate of the reactants and their proportions in order to obtain the ideal doping ratio. This concept of chemically doping a graphene layer is found extensively in biopolymer applications for energy storage due to improvements in relevant properties such as conductivity and surface area for electrodes [112]. Other carbonization methods include hydrothermal treatment that utilizes high temperature and water to decompose biopolymers and generate a carbonized scaffold. Recently, the use of KOH as a chemical activator was found to be beneficial in creating N-doped materials [113]. Another carbonization method is pyrolysis, one of the most common and direct options. Here, the carbonization temperature is optimized in the absence of oxygen to decompose the biopolymer and yield an N-doped carbon material [113].

### 4.4. Three-Dimensional Printing

**Figure 4:**
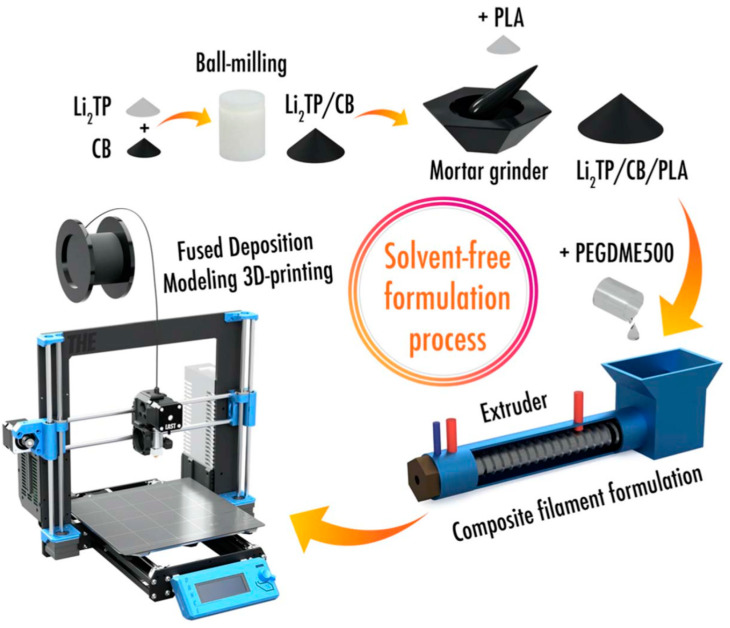
Solvent-free formulation process of a corn-based PLA-containing filament for the 3D printing of a lithium-ion battery [117]. Reproduced with permission (Copyright 2021 The Electrochemical Society, under CC BY 4.0).

Three-dimensional printing of biopolymers is an emerging method for synthesis of bio-based nanostructures, capable of producing a variety of materials including plastic blends and hydrogels [114,115]. Biopolymers are incorporated into electrolyte mixtures to be print batteries with optimal material properties. For example, biocompatible polylactic acid (PLA) was infused with a mixture of ethyl methyl carbonate, propylene carbonate, and LiClO4 to develop a high-powered lithium-ion battery [116]. Lithium-terephthalate/polylactic acid (Li_2_TP/PLA) composite filament has been used to 3D print a lithium-ion battery electrode [117]. The biopolymer can be incorporated with a solvent-free method by ball-milling and grinding the powered materials together (Figure 4). To increase sustainability, a corn-based PLA polymer matrix was also utilized for the 3D printing of materials [117].

### 4.5. Characterization of Biopolymer-Based Energy Products

An important step in the fabrication process of biopolymer-based energy products is ensuring that the desired material properties are achieved in the final product. There are a wide range of methods utilized to characterize samples after the fabrication process. Field emission scanning electron microscopy (FESEM) is often used to check the structural stability and morphologies of biopolymers after they are implemented into battery-based materials [118]. This enables researchers to specifically visualize the porosity of the membranes and/or other components. X-ray photoelectron spectroscopy (XPS) is another useful technique to help determine the chemical makeup of the final product, which is especially important for heteroatom doped scaffolds [112]. Raman spectroscopy can be utilized for a similar purpose for characterization of doped materials through analysis of the D, G, and 2D band peaks. This can be accomplished via comparisons of the defects on surfaces since doped materials often possess smaller crystallites [112]. Differential scanning calorimetry (DSC) and thermogravimetric analysis (TGA) are used to test the thermal transitions and the thermal degradations of the final product. Stress–strain mechanical test and nanoindentation are used to test the bulk and nanoscale materials strength after construction. To test for the efficacy and cycle performance of the battery, electrochemical impedance spectroscopy and cyclic voltammetry are often used to determine impacts of individual components of the energy storage device [118]. Samples are also tested for biodegradation, structure (X-ray diffraction and Fourier transform infrared spectroscopy), and morphology (atomic force microscopy, scanning electron microscopy, and transmission electron microscopy) at various scales [119].

## 5. Applications

### 5.1. Lithium-Based Batteries

Applications of biopolymers in lithium–sulfur-based batteries have become of recent interest due to their high energy density (about 2600 W·h·kg^−1^) [120]. Incorporation of carbon-based, fibrous biopolymers are advantageous for lithium–sulfur batteries due to strong physical and chemical adsorptions, low-cost, and environmental friendliness (Table 2). The resultant carbon host (i.e., the fibers) allows for high sulfur loading and fast electron transfer. Tao et al. developed carbon nanoflakes modified with metal oxides using a simple carbonization step [121]. Using a capillarity, the carbon is incorporated by first taking a solution containing metal nitrate and introducing it to the biopolymer fibers. Synthesis of a flexible carbon–cotton cathode occurs via carbonization in an argon environment at 900 °C for 6 h. This synthesized cotton carbon fiber exhibits the high porosity and surface area of this material. However, other materials besides cotton may be used for this process, including bacterial cellulose, cotton cloth, and silk and cobweb [122,123,124,125].

**Figure 5:**
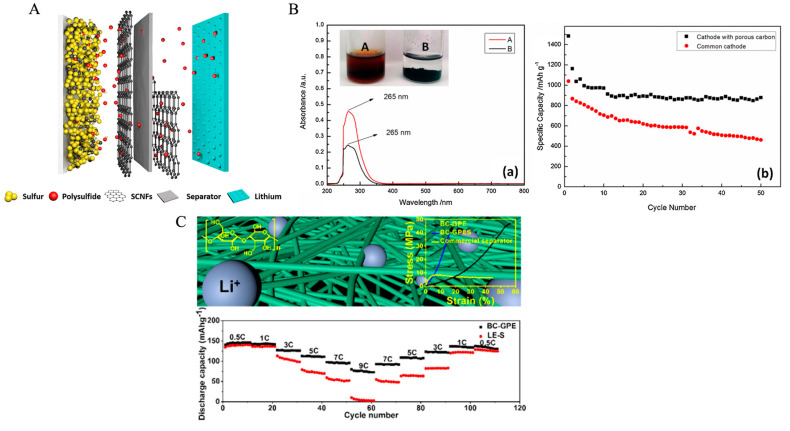
Various biopolymers utilized for synthesis of lithium-based energy storage devices. (**A**) Carbonized silk layer for lithium battery separators to reduce polysulfide transport [93]. (**B**) Porous carbon scaffold derived from fish collagen for lithium battery cathodes to extend battery performance by (**a**) reduced polysulfide absorption and (**b**) increased specific capacity [124]. (**C**) Cellulose gel for lithium battery electrolyte to limit anode dendrite growth and promote battery function [125]. Reproduced with permissions ((**A**). Copyright 2019 Elsevier; (**B**). Copyright 2014 Elsevier; (**C**). Copyright 2018 American Chemical Society).

Incorporation of materials such as soy protein, chitosan, cellulose, and fungus may also be used to boost lithium batteries [126]. Guo et al. recently proposed synthesis methods utilizing carbon-based hydrogels, which utilizes carbon-based materials such as gelatin [127]. Likewise, carbonization of silk serves as a primary application for energy storage in lithium-based batteries. Hou et al. utilized ferric chloride and zinc chloride as dissolving agents to induce a porous microstructure into silk for the electrolyte/electrode interface. They cite benefits of this as enabling quicker ion transport via the porous architecture enabling effective diffusion. Similarly, the addition of these micro- and meso-pores increases the surface area of the scaffold, also promoting electrical site availability. Overall, their novel hierarchical porous nitrogen-doped carbon nanosheets (HPNC-NS) displays a reversible lithium-ion storage capacity of 1865 mA·h·g^−1^ and an energy density of 102 W·h·kg^−1^ suggesting promise for future use to improve battery and supercapacitor’s function [92]. As seen in Figure 5, Wu et al. also used electrospun silk in lithium–sulfur batteries to increase battery stability. They explained that Li–S batteries suffer from the “shuttle effect”, characterized by dissolution of the polysulfides through the separator between the electrodes [93]. Here, polysulfides react with the lithium anode via migration through the electrolyte and break into shorter fragments. These same fragments can return to the sulfur cathode and reform the original polysulfide, creating a cyclic tendency hindering battery function. To target this inefficiency, Wu et al. synthesized a carbonized regenerated silk nanofiber (SCNF) to function as an interlayer covering both sides of the separator. They found that this new battery structure maintained high energy capacity and limited charge dissipation [93]. Song et al. modified this approach, functionalizing the interlayer with both nitrogen and phosphorus (N/P) to trap polysulfides via charge and ultimately stabilize the cell. Overall, this design also demonstrated high performance and low decay in cyclic examinations [128].

Moreover, keratin has also been identified as another natural source for enhancing lithium battery function. Thiyagarajan et al. detailed more of the pitfalls of lithium-ion batteries including a low working potential and deposition caused by current anode material selections [94]. They justify using TiNb_2_O_7_ (TNBO) due to its high capacity and working potential, though this material lacks proper ionic conductivity and electronic properties. To adjust for this, keratin was introduced to create a carbon-rich nanocomposite for anode applications. Compared with unmodified TNBO, the nanocomposite exhibited greater ionic conductivity, higher discharge capacity, and maintained an elevated reversible capacity [94]. Dong et al. utilized a similar approach in creating a different nanocomposite. Here, hematite is combined with the carbonization of hair-derived keratin in order to create a porous scaffold for lithium battery anodes [129]. As cited above, the increased porosity of the cell yields more efficient ion diffusion, in addition to relieving volume expansion issues caused by lithium usage. Overall, this battery demonstrated a reversible capacity of 1000 mA·h·g^−1^ after 200 cycles and a discharge capacity of 750 mA·h·g^−1^ at 2 C, which was better performance than simply using hematite as the anode [129].

The sheer abundance of collagen enables a unique level of application into energy storage. Many groups have attempted to repurpose waste sources of collagen for electrical materials. For example, to tackle issues with polysulfide dissolution in lithium–sulfur batteries, causing anode corrosion, Wei et al. utilized tissue from pig bone (highly composed of collagen fibers) as a bone-based hierarchical porous carbon (BHPC) scaffold for cathode applications [130]. Due to this porous microarchitecture and resultant increased surface area, the subsequent lithium–sulfur batteries portrayed sufficient conductivity as well as sustained cycle efficiency after 50 cycles, dropping from 1265 mA·h·g^−1^ to 643 mA·h·g^−1^ [130]. Gao et al. utilized fish-based collagen to specifically target the “shuttle effect” plaguing lithium sulfur batteries [124]. As an additive, their sulfur/carbon nanocomposite demonstrates increased absorption of polysulfides, as confirmed by UV spectroscopy in Figure 5 and SEM, thus preventing the repetitive movement. The researchers reveal not only an increased specific capacity of the cathode, but also higher retention and reversible capacity after 100 cycles [124]. Odoom-Wubah et al. purified collagen from local market fish waste and utilized it as an anode material for various batteries, namely the lithium-ion class. The fish collagen was doped with lead nanoparticles to fulfill its original function as a catalyst for benzene oxidation; however, the material demonstrated excellent electrochemical properties including a specific capacity of 270 mA·h·g^−1^ [97].

As suggested by Song et al., the presence of nitrogen can be beneficial in lithium batteries to trap polysulfides and prevent the shuttle effect, which leads to inefficiencies. Chen et al. attempted a similar method using chitosan as an additive for both the cathode and separator of a lithium–sulfur battery [101]. The researchers note chitosan’s viscosity as another factor for its ability to encapsulate these moving polysulfides. Ultimately, inserting a chitosan membrane in the cell enabled both a high discharge capacity and improved reversible specific capacity in comparison with an uncoated lithium–sulfur battery [101]. Previous works have also successfully modified the microarchitecture of chitosan to induce a porous structure, such as the effect achieved by electrospinning silk. Alias et al. found that dissolving out silicon dioxide from chitosan (through solution cast and porogen elimination) ameliorated several electrochemical properties in a coin cell proton battery such as conductivity and specific discharge capacities [131]. This likely can be attributed to a less hindered path for charges to travel in the cell.

Cellulose, the most abundant biopolymer in the world, presents a cost-effective option for energy solutions. Polyolefins are the current standard used as separators for lithium-ion batteries, but they have non homogenous porosity, low dimensional stability, and their thermal shrinkage puts these batteries at risk of short circuiting [132]. Gonçalves et al. designed a single polymer cellulose membrane fabricated of mesoporous cellulose nanocrystals and an ionic liquid electrolyte [104]. To further optimize the properties of a cellulose membrane, Xu et al. created a composite composed of bacterial cellulose and Al_2_O_3_ with improved capacity, conductivity, and uptake [133]. Additionally, cellulose membranes can improve other aspects of lithium-ion batteries. For instance, Xu et al. developed a bacterial cellulose-based gel polymer electrolyte able to limit dendrite growth at the lithium anode as found in Figure 5 [125]. Song et al. used a gel polymer electrolyte fabrictaed of lignocellulose from treated wood which demonstrated improved rate and cycling performance by limiting the shuttle effect [134].

Additionally, agarose presents an interesting opportunity for lithium-ion battery development. The development of silicon anodes in lithium-ion batteries has been limited by low conductivity and decreased capacity with cycling [135]. To overcome this, anode binders have been investigated with agarose as a promising solution. Hwang et al. found an agarose binder on silicon led to increased electrochemical properties and retained charge capacity with cycling [88]. Lithium dendrite formation is a serious challenge facing the further adoption of lithium-based anodes; Zhang et al. developed an agarose film which prevented lithium dendrite formation and the resulting malefices [136]. Li/LiMn_2_O_4_ cells with polyethylene separators are limited by a loss of electrochemical stability within 45 cycles due to Mn^2+^ ion deposition on the anode or precipitation on the electrode [105]. Kim et al. mixed polyacrylonitrile, polyacrylic acid, and agarose as a separator in a Li/LiMn_2_O_4_ cell which captured Mn^2+^ ions allowing for continued electrochemical stability after 100 cycles.

**Table 2:** Biopolymer applications as lithium battery components.

Application	Function	Initial Reversible Capacity	Coulombic Efficiency	Cycling Stability
Silk-derived hierarchical porous nitrogen-doped carbon nanosheets [92]	Anode in Li-ion	1913 mA·h·g^−1^ at 0.1 A·g^−1^	49.2% at 0.1 A·g^−1^	9% loss after 10,000 cycles
Carbonized silk fibroin nanofiber film [93]	Cathode and anode interlayers in Li/S	1164 mA·h·g^−1^ at 0.2 coulomb (C)	97.3% at 1.0 C	69% retention after 200 cycles
Silk-derived N/P co-doped porous carbon mixed with sulfur [128]	Cathode in Li/s	888.5 mA·h·g^−1^ at 1.0 C	97.6% at 1.0 C	0.032% loss per cycle over 500 cycles at 1.0 C
Keratin-derived carbon added to TiNb_2_O_7_ [94]	Anode in Li-ion	356 mA·h·g^−1^ at 0.1 C	55.0% at 0.1 C	85% retention after 50 cycles at 1 C
Keratin-derived carbon combined with α-Fe_2_O_3_ nanoparticles [129]	Anode in Li-ion	1690 mA·h·g^−1^ at 0.2 C	75% at 0.2 C	Capacity of 1000 mA·h·g^−1^ at 0.2 C after 200 cycles
Collagenous bone-based hierarchical porous carbon combined with sulfur [130]	Cathode in Li/S battery	1265 mA·h·g^−1^	–	Capacity of 643 mA·h·g^−1^ after 50 cycles
Collagenous hierarchical porous carbon added to sulfur [124]	Cathode in Li/S battery	1426 mA·h·g^−1^	Greater than 98% at 1 C after 100 cycles	81% retention after 50 cycles at 1 C
Collagen doped with Pd/PdO nanoparticles [97]	Anode in Li/S battery	276 mA·h·g^−1^	–	200 mA·h·g^−1^ after 20 cycles
Chitosan combined with sulfur and separately chitosan combined with carbon [101]	Cathode and separator, respectively in LiS	1145 mA·h·g^−1^	98% atvarious cycling rates after 100 cycles	Capacity of 646 mA·h·g^−1^ after 100 cycles at 1 C
Mesoporous cellulose nanocrystal membrane [104]	Membrane for Li-ion	122 mA·h·g^−1^ at C/2	Nearly 100% after initial decay	Retention above 90% up to C
Bacterial cellulose combined with Al_2_O_3_ in a membrane [133]	Separator for Li-ion	161 mA·h·g^−1^ at 0.2 C	–	89% retention after 50 cycles at 0.2 C
Cross linked bacterial cellulose gel [125]	Electrolyte for Li-ion	141.2 mA·h·g^−1^ at 0.5 C	89.46% at 0.5 C	104.2% retention after 150 cycles at 0.5 C
Lignocellulose-based gel [134]	Electrolyte for LiS	1186.3 mA·h·g^−1^ at 20 mA·g^−1^	–	55.1% capacity retention after 100 cycles at 20 mA·g^−1^
Hard carbon from agarose [88]	Binder for LiMn_2_O_4_ cathode	101 mA·h·g^−1^ at 0.05 C	~96.2% at 0.05 C	~100% retention after 400 cycles at 0.2 C
Uniform agarose film combined with copper foil [136]	Protective layer for Li anode	117.1 mA·h·g^−1^ at 1.75 mA·cm^−2^	96% at 4 mAh cm^−2^	87.1% retention after 500 cycles at 1.75 mA·cm^−2^

### 5.2. Zinc-Based Batteries

**Figure 6:**
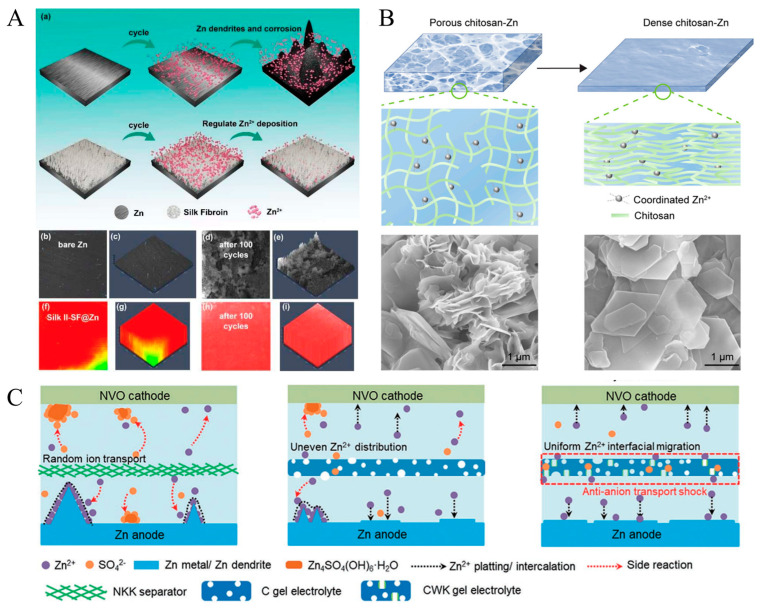
Various biopolymers utilized for fabrication of zinc-based batteries. (**A**) Utilization of silk fibroin as a protective layer for zinc anode to (**a**) prevent dendrite formation as seen by two-photon excitation microscopy of (**b**–**e**) zinc and (**f**–**i**) silk coated anode [137]. (**B**) Fabrication of unique and tunable polymer gel electrolyte with chitosan in both (**a**) porous and dense form with respective (**b**,**c**) SEM images to limit dendrite formation [138]. (**C**) Incorporation of recycled keratin for electrolyte gel that induces porosity in battery cells to facilitate better ion transport and distribute potential dendrite growth [95]. Reproduced with permissions ((**A**). Copyright 2022 John Wiley and Sons; (**B**). Copyright 2022 Elsevier; (**C**). Copyright 2022 John Wiley and Sons).

Moreover, zinc metal batteries are another class of electrochemical materials that have a promising future due to rechargeability, excellent energy density, cheap cost, lower environmental footprint, and scalability. However, they often fail ultimately due to zinc deposition caused by the aqueous electrolyte that reacts with zinc. In addition, formations of dendrites hinder the potential of zinc-based batteries, resulting in unwarranted contact between the cathode and electrode that causes corrosion. To tackle such issues for an otherwise promising class of sustainable batteries, biopolymers can be incorporated to limit undesirable interactions (Table 3). For example, Lu et al. utilize silk fibroin as a protective layer for aqueous zinc ion batteries (AZIB), specifically for the zinc anode which is where the dendrites typically originate [137]. They note the importance of purifying silk to isolate silk II (primarily β sheets) that limit unwanted reactions with the mildly acid electrolyte and restrict side reactions with water due to hydrophobicity [137]. As depicted in Figure 6, Lu and coworkers found that coating the zinc anode with this purified silk enabled a more uniform deposition of zinc ions onto the anode due to polar amino acids in the biopolymer, reducing the formation of dendrites. The battery can operate for over 3300 h at 10 mA·cm^−2^ and was demonstrated to have superior performance in comparison with a battery without the silk coating [137]. One growing area of interest in battery development is the fabrication of potent transient batteries characterized by their degradation over a set time period. These batteries possess unlimited application in various medical, military, and commercial products. Zhou et al., developed a gelatin–silk composite film as the electrolyte for a transient zinc-based battery. Gelatin, a denatured form of collagen, served as a humidity dependent structure due to its thermo-reversible properties [98]. The addition of gelatin to silk fibroin enables faster plasticization of the polymer. Overall, the gelatin–silk biopolymer composite for the electrolyte allowed for a specific capacity of 311.7 mA·h·g^−1^, retained almost 95% of capacity after 100 cycles, and predictably degraded after 45 days [98].

Keratin has also been briefly utilized for zinc-based batteries. Shao et al. delineated their carrageenan and wool keratin bio gel that would be used as the electrolyte for their cell [95]. As mentioned above, the use of a polymer gel poses numerous advantages if optimized. As illustrated in Figure 6, Shao and coworkers explain that their hybrid gel not only provides a porous matrix to facilitate better ion movement, but also stabilize the zinc charge through the charges found on residues of keratin in order to prevent dendrite formation and ultimately unwanted side reactions between the electrode and electrolyte [95].

The application of chitosan-based batteries also goes beyond the lithium-ion class. Poosapati et al. demonstrated the efficacy of a chitosan–PVA gel to serve as a polymer electrolyte for a zinc rechargeable battery [102]. The previous literature had not portrayed the efficacy of a non-aqueous electrolyte for this purpose and required a cathode additive. Utilizing such a biopolymer electrolyte is highly sustainable in the case of battery leakages and disposals. Overall, Poosapati et al. marked the performance and reversibility of these novel zinc-electrolytic manganese dioxide batteries with chitosan as on par to those batteries without the chitosan additive [102]. Wu et al. further characterized and improved upon the issues present within the highly promising zinc metal rechargeable batteries in Figure 6. They fabricated a chitosan-Zn^2+^ polymer gel through coordinate covalent bonding and mechanically modulated its water content to create a dense network. Researchers found that such alterations not only exhibited excellent reversibility and cycling stability, but also the prevention of dendrite formation [138]. Another important aspect of batteries targeted via chitosan is the separator, which is typically found in the electrolyte. Here, Yang et al. modified the traditionally used filter paper with a chitosan coating to harness the beneficial properties of this biopolymer in order to ameliorate some common issues in zinc ion batteries. Namely, Yang and colleagues explained how the oxygen-rich amino acids in chitosan can interact with protons in the interact, thereby limiting their activity and preventing unwanted side reactions usually causing corrosion [139]. As seen with silk, this chitosan coating also promoted the more homogeneous adsorption of zinc ions, restricting the degree of dendrite formation [139]. Overall, this zinc-ion battery with chitosan filter paper almost doubles the capacity retention in comparison with a control cell and is effective for 2900 h [139].

### 5.3. Capacitors

**Figure 7:**
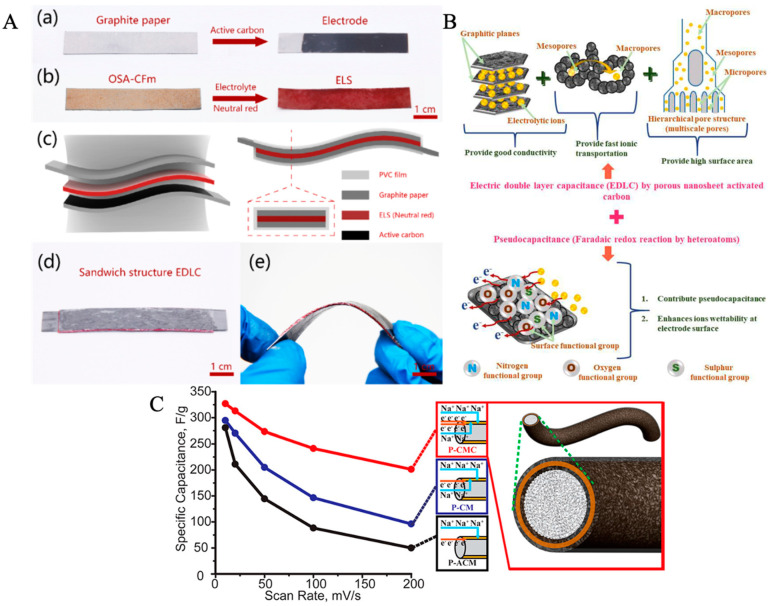
Various biopolymers utilized for fabrication of capacitors. (**A**) Synthesis of novel locked collagen separator with unique (**a**–**d**) sandwich structure, composed of electrolyte and electrode, and (**e**) bending ability as material for supercapacitors to better interface electrolyte and porous scaffold [100]. (**B**) Utilization of keratin from human hair for electrodes of capacitor in order to enhance specific capacitance [67]. (**C**) Application of hydrophilic cellulose properties in a film (paper/CNTs/MnO_2_/CNTs (P-CMC), paper/CNTs/MnO_2_ (P-CM), or paper/Al_2_O_3_/CNTs/MnO_2_ (P-ACM)) to guide electron movement in electrolyte [141]. Reproduced with permissions ((**A**). Copyright 2020 Elsevier; (**B**). Copyright 2020 Elsevier; (**C**). Copyright 2013 American Chemical Society).

Similar to batteries, biopolymers generally amplify electrical properties in traditional electrode materials for capacitors (Table 4). Capacitors are an alternative energy storage solution that benefit from faster charging; however, they demonstrate limitations in energy density [96]. Supercapacitors are a subset of capacitors that display properties of both a capacitor and store energy within the electrolyte, such as a battery [96]. Subhani et al. demonstrated a method to repurpose collagen from poultry waste for capacitor electrodes. In this study, the researchers developed a porous collagen scaffold with graphene nanoparticles to increase conductivity. They claimed that such modifications enable more efficient charging and discharging of the capacitor due to increased surface area (creating higher ion adsorption) and better ion transport due to enhanced porosity and stable 3D microarchitecture [140]. In summary, this sponge-like electrode achieved a higher capacitance (365 F·g^−1^) than competing biopolymers such as keratin and a cyclic stability of 97% at 100 mV·s^−1^ after 10,000 cycles [140]. Lee et al. shared another approach for fabricating capacitor electrodes by synthesizing carbon nanosheets through carbonization of collagen. In this work, the researchers sought to develop a material for electric double-layer capacitors (EDLCs). Like the rationale for batteries, the doping of a nitrogen-rich substance (such as collagen via amino groups) adds electronic functionality to the material [99]. This was quantified via the oxygen reduction reaction (ORR). In the end, this collagen-based carbon nanosheet demonstrated greater ORR compared with an industry standard 20% Pt electrocatalyst and had an 80% capacitance retention [99]. In addition to the electrode, collagen is effective as a separator for supercapacitors. In this case, Xu et al. utilized a collagen and sodium alginate composite as a scaffold for the separator in an electric double-layer capacitor. They emphasized the high conductivity of liquid electrolytes, but also acknowledged the possibility of leakages that typically plague them [100]. Therefore, Xu and coworkers claimed that this novel separator, shown in Figure 7, can “lock” the electrolyte due to the porous structure of the collagen membrane that is further chemically crosslinked with sodium alginate [100]. Ultimately, through a proof of concept, the electrolyte-locked separator capacitors exhibited comparable performance to a standard cellulose separator sharing the potential for fabricating a hybrid separator that enables the benefits of both solid state and liquid electrolytes [100].

Keratin also possesses similar capabilities as collagen for capacitor applications. Ramakrishnan et al. developed an activated, porous carbon scaffold from goat keratin for the cathode of the capacitor [96]. In a sodium hybrid capacitor, the capacitor portrayed a capacitance of 256 F·g^−1^ and a power density of 95 W·kg^−1^, though retaining of the initial capacitance was slightly limited at 78% [96]. Sinha et al. also attempted to create a novel material for the electrodes of EDLCs. They used keratin derived from human hair to synthesize their carbon nanosheet, citing the abundance of nitrogen, oxygen, and sulfur as advantageous for ion adsorption and movement [67]. Specifically, oxygen from carboxyl and carbonyl groups in keratin can enhance the pseudo capacitance of the supercapacitor [67]. Overall, this keratin-derived carbon nanosheet boasts excellent performance such as a specific capacitance value of 999 F·g^−1^, energy density of 32 W·h·kg^−1^, and 98% retention of capacitance after 10,000 cycles as seen in Figure 7 [67]. Sinha et al. builds on this previous work using an asymmetric capacitor, essentially denoting the presence of two distinct electrodes in the system [142]. Once again, the keratin-derived porous carbon sheets are used for the cathode. Here, they find that the keratin-derived carbon yields a specific capacitance of 560 F·g^−1^ and a rate capability of 40% until 40 A·g^−1^ is reached [142]. Wu et al. also shared a similar approach for a keratin-based electrode for capacitors. They derive keratin from poultry, citing the large wastages from the industry. As seen above, the addition of a biopolymer such as keratin adds surface area to the electrodes and creates a porous structure, enabling ion transport [143]. The presence of atoms such as nitrogen also promotes stability of the capacitor. In the end, this capacitor showed a capacitance of 270 F·g^−1^ and retained a capacity of up to 98% after 10,000 cycles [143].

Cellulose also offers an opportunity for the development of supercapacitors. One method of using cellulose is taking advantage of its insulating capabilities to help direct charge flow [144]. Liew et al. used this concept to make a cellulose nanocrystal backbone and polypyrrole film which demonstrated an improved capacitance and pore network over state-of-the-art electrode films fabricated of polypyrrole and carbon nanotubes [103]. Another method is to use the hydrophilic nature of cellulose with a liquid electrolyte to guide ion transport. Gui et al. developed a film allowing for dual electron and ion transport fabricated of cellulose paper dipped in carbon nanotubes with MnO_2_ as an active material, with the entire structure then dipped again in carbon nanotubes as depicted in Figure 7 [141].

##  6. Conclusions, Challenges, and Future Perspectives

Biopolymers offer an exciting new area for development in energy storage materials. There exists a wide variety of biopolymers suited to different roles in energy storage apparatus based on their unique characteristics, each can be an ingredient capable of addressing challenges with existing energy storage devices. The advantages shared by natural biopolymers are their biodegradability, biocompatibility, and renewability. The two main classes of biopolymers used in these applications explored in this review are polypeptides (silk, keratin, and collagen) and polysaccharides (chitosan, cellulose, and agarose).

To create effective energy storage devices, an understanding of the electricity laws and theorems helps guide the understanding of the various roles and their requirements. Qualities of note for the device include total capacity, capacity after repeated charge cycles, and for capacitors, capacitance. The main roles needed for energy storage applications are an anode, a cathode, an electrolyte, a separator, and a current collector. When creating the energy storage device, understanding the underlying theory allows for optimization in terms of these roles to uniquely fit the requirements needed.

Several techniques exist for the fabrication of these biopolymers into forms suitable for energy applications. Solution casting is an effective technique to create membranes suitable for biopolymer electrolyte membrane applications. Electrospinning is a common and effective method of manufacturing biopolymers due to the amount of control a manufacturer maintains over the product, creating a highly tunable nanofiber that can be created of various types of materials. Three-dimensional printing allows for a distinct level of control in the final structure; ball milling, grinding, and extrusion allow for creation of a filament to be used by the 3D printer. After using one of several fabrication methods, testing is paramount to ensure the desired proper structure and properties.

A wide variety of biopolymers find use as various components of these batteries to improve total capacity, energy density, or prevent dendrite formation. Lithium-ion batteries are the current standard for rechargeable energy storage solutions, but biopolymer-based materials offer an avenue for improvement. Zinc batteries are a promising alternative to lithium-ion batteries with lower environmental and financial cost but are limited due to zinc deposition and dendrite formation. Silk, keratin, and chitosan all have promising applications to curtail dendrite formation causing cathode and electrode contact. Capacitors, specifically supercapacitors, have a promising future in energy storage due to increased chagrin capacity; however, this typically comes at a cost to total energy density. In this application, collagen, keratin, and cellulose are exciting biopolymers for capacitor optimization, offering decreased charging rates due to a porous separator structure. In addition to the total theoretical energy density, other challenges faced by biopolymers in electrical solutions include the cost required to effectively manufacture such parts, their shelf life, and lack of understanding of if such a concept can consistently outperform industry standard options. Therefore, the outlook remains to further advance biopolymer science in energy storage applications ideally leading to a fully biodegradable product.

Integrating components fabricated of biopolymer-based materials into energy storage devices allow for taking advantage of their individual natural properties, low environmental impact, as well as their ease in incorporation with other conductive materials [141,142]. As improved energy storage solutions are continuously demanded, biopolymers offer a promising path for their development.

## Figures and Tables

**Table 3:** Biopolymer applications as zinc battery components.

Material	Function	Initial Reversible Capacity	Coulombic Efficiency	Cycling Stability
Silk II–silk fibroin [137]	Coating for Zn anode	189 mA·h·g^−1^ at 0.1 A·g^1^	As high as 99.7%	As long as 3300 h at 10 mA·cm^−2^ and 10 mA·h·cm^−2^
Gelatin–silk protein film [98]	Electrolyte film	311.7 mA·h·g^−1^	Greater than 90% over 100 cycles	Greater than 90% over 100 cycles
Carrageenan and wool keratin bio gel [95]	Electrolyte film	271.6 mA·h·g^−1^ at 0.1 A·g^1^	~98%	96% capacity retention after 500 cycles
Chitosan-based gel electrolyte with poly(vinyl alcohol) added [102]	Electrolyte film	310 mA·h·g^−1^ at 0.1 A·g^1^	96.5% at 0.5 A·g^−1^	~70% capacity retention after 300 cycles
A sustainable chitosan-zinc electrolyte for high-rate zinc metal batteries [138]	Electrolyte	208 mA·h·g^−1^	99.7%	Greater than 400 cycles
Chitosan modified filter paper [139]	Separator	323 mA·h·g^−1^ at 0.1 A·g^1^	99.6% at 1 mA·cm^−2^ and 1 mA·h·cm^−2^	98.4% retention over 1000 cycles

**Table 4:** Biopolymer applications as capacitor components.

Material	Function	Capacitance	Surface Area	Cycling Stability
Graphene-integrated porous carbon derived from collagen [140]	Electrode	365 F·g^−1^ at 1 mV·s^−1^	1087 m^2^·g^−1^	97% capacitance retention after 10,000 cycles at 100 mV·s^−1^
N-doped carbon nanosheets from collagen cross linked with paraformaldehyde [99]	Electrode	102 F·g^−1^ at 25 mV·s^−1^	695 m^2^·g^−1^	80% capacitance retention at 1000 mV·s^−1^
Collagen fiber membrane cross linked with oxidized sodium alginate [100]	Electrolyte locked separator	143.07 F·g^−1^ at 5 mV·s^−1^	–	99.99% capacitance retention after 10,000 cycles at 10.0 A·g^−1^
Keratin-derived reduced graphene oxide combine with MoO_2_ [96]	Electrode	256 F·g^−1^ at 0.02 A·g^−1^	2042 m^2^·g^−1^	86% capacitance retention after 1000 cycles at 0.07 A·g^−1^
Keratin-derived carbon nanosheets [67]	Electrode	999 F·g^−1^ at 1 A·g^−1^	1548 m^2^·g^−1^	98% capacitance retention after 10,000 cycles at 5 A·g^−1^
Keratin-derived redox active carbon [142]	Electrode	560 F·g^−1^ at 1 A·g^−1^	1483 m^2^·g^−1^	95% capacitance retention after 10,000 cycles at 5 A·g^−1^
Carbonized keratin combined with H_2_SO_4_ [143]	Electrode	270 F·g^−1^ at 1 A·g^−1^	2684 m^2^·g^−1^	98% capacitance retention after 10,000 cycles at 10 A·g^−1^
Nanocomposite film formed from cellulose nanocrystals and polypyrrole [144]	Electrode	336 F·g^−1^	–	Limited swelling after 5000 cycles
Cellulose paper integrated with carbon nanotubes and MnO_2_ [141]	Electrode	327 F·g^−1^ at 200 mV·s^−1^	–	62% capacitance after 12,500 cycles

## Data Availability

Not applicable.
